# The Biological Relevance of NHERF1 Protein in Gynecological Tumors

**DOI:** 10.3389/fonc.2022.836630

**Published:** 2022-02-11

**Authors:** Margherita Sonnessa, Sara Sergio, Concetta Saponaro, Michele Maffia, Daniele Vergara, Francesco Alfredo Zito, Andrea Tinelli

**Affiliations:** ^1^ Functional Biomorphology Laboratory, Pathology Department, IRCCS Istituto Tumori “Giovanni Paolo II”, Bari, Italy; ^2^ Department of Biological and Environmental Sciences and Technologies (DiSTeBA), University of Salento, Lecce, Italy; ^3^ Department of Obstetrics and Gynecology, “Veris delli Ponti” Hospital, Lecce, Italy

**Keywords:** NHERF1, gynecological cancers, cervical cancer, ovarian cancer, Wnt/beta-catenin pathway

## Abstract

Gynecological cancer management remains challenging and a better understanding of molecular mechanisms that lead to carcinogenesis and development of these diseases is needed to improve the therapeutic approaches. The Na^+^/H^+^ exchanger regulatory factor 1 (NHERF1) is a scaffold protein that contains modular protein-interaction domains able to interact with molecules with an impact on carcinogenesis and cancer progression. During recent years, its involvement in gynecological cancers has been explored, suggesting that NHERF1 could be a potential biomarker for the development of new targeted therapies suitable to the management of these tumors. This comprehensive review provides an update on the recent study on NHERF1 activity and its pathological role in cervical and ovarian cancer, as well as on its probable involvement in the therapeutic landscape of these cancer types.

## Gynecological Cancers: Emerging Molecular Aspects

Gynecological cancers include a diverse group of neoplasms that originate in a woman’s reproductive system. This includes tumors that originate from these distinct anatomical sites including vulva, vagina, corpus uteri, fallopian tube and ovary (1). These tumor types represent an important cause of cancer morbidity and mortality worldwide.

Recent findings have defined the molecular profile of gynecological tumors at a systemic level. In this scenario, a work published in 2019 comprehensively defined the mutational, gene and protein expression profile of a cohort (Pan-Gynecologic from the The Cancer Genome Atlas) of over 2000 cases of four gynecological types plus breast (2). Results of this study demonstrated that pan-gyn tumors are unique compared to other tumor types and result from the interplay of different factors including multiple genomic and epigenomic features and somatic copy-number alterations. Among the pan-gyn tumors, studies of expression profiles suggest that at least nine and five clusters with distinct clinicopathologic characteristics can be observed at the mRNA and protein level, respectively.

The results of a subsequent study conducted on a cohort of 209 patients diagnosed with cervical, endometrial and ovarian cancer further defines the molecular differences and similarities between these different cancer types and assessed whether molecular heterogeneity is observed in an early or late stage of tumorigenesis (3). The results of this study led to two important conclusion: i) the identification of a common reprogramming process emerging at the early stages of tumorigenesis which involves the activation of phosphatidylinositol 3-kinase (PI3K) protein, defects in mismatch repair program and cilium organization, as well as disruption in interferon signaling and immune recognition; ii) a cell-type specific program that is activated at a late-stage tumor development.

**Figure 1:**
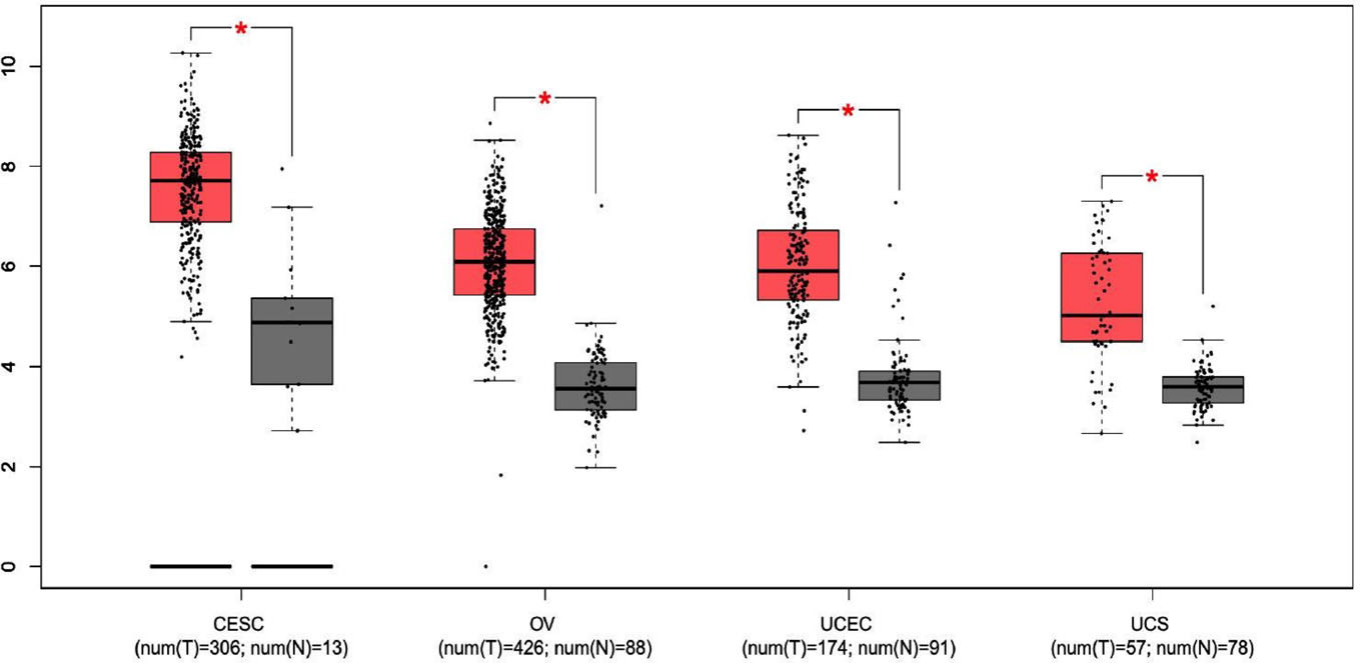
The expression of NHERF1 protein in different gynecological tissues. Boxplot results of the expression levels of NHERF1 in cervical squamous cell carcinoma and endocervical adenocarcinoma (CESC), ovarian cancer (OV), uterine corpus endometrial carcinoma (UCEC) and uterine carcinosarcoma (UCS) analyzed using GEPIA database. Red box, tumor samples; green box, normal samples. T, tumor; N, normal. P-value was set up at 0.01. (* = p < 0.01).

Although gynecological tumors emerge from a common precursor (coelomic epithelium, also known as mesothelium), data from these studies demonstrate the presence of molecular pathways relevant to sustain this tumor heterogeneity useful for diagnostic and therapeutic purposes. Central nodes include PI3K, AKT, and Wnt-β-catenin pathways among the others, that are involved in the regulation of biological processes such as cell cycle, motility, metabolism, and differentiation. In pan-gyn tumors, the mutational landscape and expression levels of these kinases is well described but less is known about other regulatory mechanisms. For instance, their biological activity is regulated through the dynamic interactions with scaffold proteins that bind and direct the cellular localization of their interaction partners. One of these scaffold proteins with a well-established role in the regulation of cell signaling is the protein sodium/hydrogen exchanger regulatory factor 1 (NHERF1). In this review article, we describe the functional significance of NHERF1 in gynecological tumors. As it is upregulated in tumor tissues compared to normal samples (**Figure 1**), NHERF1 could represent a potential target for clinical applications.

## Structural and Functional Aspects of Na(+)/H(+) Exchange Regulatory Cofactor NHERF1 Protein

The sodium/hydrogen exchanger regulatory factor 1 (NHERF1), known also as the 50-kDa ezrin-binding protein (EBP50) comes into sight in the scientific landscape in the late 1990s as a co-regulator of the exchanger of the sodium–hydrogen antiporter 3 (NHE3) in rabbit kidney epithelia. In detail, NHERF1 is an adaptive protein, encoded by the *SLC9A31R1* gene, physiologically expressed in the sub-plasma membranous region of human tissues including, lung, gastrointestinal tract, liver and gallbladder, kidney and urinary bladder, male and female tissues (data from https://www.proteinatlas.org/ENSG00000109062-SLC9A3R1/tissue).

NHERF1 belongs to the NHERF family of protein–protein interaction modules (PDZ)-scaffold proteins. The PDZ family of proteins is one of the largest in the human proteome including membrane-associated guanylate kinases (MAGUKs), involved in different molecular signal networks (4); nitric oxide synthase 1 (neuronal) (NOS1), engaged in neurotoxicity associated with neurodegenerative diseases (5); segment polarity protein dishevelled homolog DVL1-2-3 (DVL1-2-3), implicated in cell proliferation and molecular transducer for different human diseases (6). Scaffolding proteins play a critical role in coordinating cellular response by the assembly of multiple complexes that make faster and more effective the molecular signals. Thanks to this ability, they can improve the efficiency and selectivity of intracellular signaling and have a pivotal role in oncogenic development.

**Figure 2:**
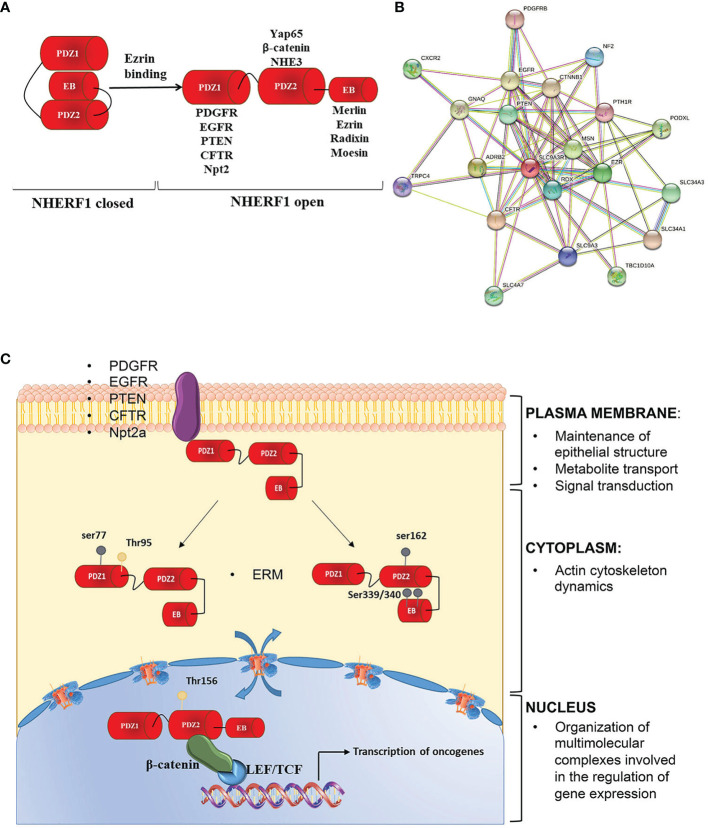
NHERF1 molecular structure and functions. **(A)** NHERF1/EBP50 presents a closed conformation in which the PDZ2 domain binds to the C-terminal EB region in a “head to tail” interaction masking the PDZ domains. Binding of the ERM proteins to the EB region switches NHERF1 to an open conformation in which the PDZ domains are unmasked and able to bind their interactive partners. **(B)** NHERF1 protein-protein interaction network, according to STRING software. **(C)** NHERF1 functions are influenced by its subcellular localization, phosphorylation and expression. NHERF1 is normally located at the plasma membrane where it has a protective role. Phosphorylation on ser 339/340 on the EB domain or on Thr 156 in PDZ2 domain increases NHERF1 binding affinity to its targets promoting its translocation in the cytoplasm where NHERF1 can have a pro-neoplastic and metastatic role and can alter integrity of actin cytoskeleton. Phosphorylation on Thr 156 causes NHERF1 nuclear localization where it is involved in regulation of oncogenes expression and cancer progression. Possible interactors of NHERF1 are also indicated.

In specific, NHERF family consists of four members including NHERF1/*SLC9A31R1*, NHERF2/*SLC9A3R2*, NHERF3/*PDZK1*, and NHERF4/*PDZD3*. Each member presents a unique molecular organization and tissue distribution. In detail, the N-terminal domain of NHERF1 is characterized by two post-synaptic density 95/disc-large/zona occludens (PDZ) repeated domains. A hallmark of these domains is the ability to target specific proteins. In fact, most NHERF1 associated proteins bind to the first PDZ domain and only a few proteins, such as NHE3 (7), β-catenin (8) and Yap 65 (9), specifically interact with the second PDZ domain. The C-terminal region is identified as an EZRIN binding domain (EB) that mediates the connection with the family of EZRIN-Radixin-Moesin (ERM) proteins (10). The EB domain ends in a PDZ motif similar to the (S/T_X_L) PDZ motif present in other NHERF1 ligands (11). The binding of EB to the PDZ2 domain of NHERF1 creates an intra-molecular interaction that induces a self-inhibited conformation status. This prevents both PDZ domains from binding their specific ligands. After the binding of ERM proteins to the EB region, the intramolecular head-to-tail conformation is lost, allowing the association among NHERF1 binding partners and PDZ domains (**Figures 2A, B**).

It has been demonstrated the ability of the two PDZ domains to regulate differently the metastatic properties of cancer cells. Indeed, the selective inactivation of one of the two domains in a model of breast cancer supports the activation of a visceral or bone metastatic dissemination route. This highlights the substantial molecular differences in the phenotypic programs regulated by the two PDZ domains (12).

NHERF1 functions as molecular scaffolds to coordinate different signaling processes. However, recent works have demonstrated that NHERF1 is more than a mere scaffold or anchor in the cell. Indeed, in a variety of diseases and tissues, NHERF1 has been shown to contribute to:

•   Cell polarity and regulation of actin cytoskeleton dynamics, through the interaction with ERM proteins and β-catenin;
•  Metabolite transport at the cell membrane, through the interaction with the type 2a sodium-phosphate cotransporter (NPT2a) and the cystic fibrosis transmembrane conductance regulator (CFTR);
•   Signal transduction, through the interaction with the protein phosphatase and tensin homolog (PTEN), the platelet-derived growth factor receptor (PDGFR) and the epidermal growth factor receptor (EGFR);
•   Gene transcription, through the interaction with β-catenin.

These different functions can be altered by NHERF1 localization, phosphorylation and expression (**Figure 2C**) (13).

When NHERF1 is localized at the plasma membrane, the protein preserves the epithelial polarity through the formation of tight junctions (14), targets several proteins to the apical or basolateral membrane (15, 16) and cooperates in the formation and assembly of protein complexes at the microvillus (17). Instead, when located within the cell NHERF1 reduces the integrity of the actin cytoskeleton, by increasing α-actinin IV ubiquitination and degradation (18).

The localization and oligomerization, as well as the assembly and disassembly from protein complexes of NHERF1 are regulated through phosphorylation. In detail, phosphorylation on human serine 339/340 (serine 337/338 in rat) favors NHERF1 oligomerization by increasing PDZ2 accessibility and NHERF1 binding affinity to its targets (19), while phosphorylation on other sites influences NHERF1 subcellular localization. Indeed, it has been observed that NHERF1 phosphorylation on human serine 339/340 by protein kinase C (PKC) induces NHERF1 relocalization from cell periphery to the cytosol (20). Phosphorylation at serine 77 the PDZ I domain impairs NHERF1 affinity for plasma membrane proteins thus influencing its subcellular localization (21). Although NHERF1 does not present a nuclear localization signal, it has been found in the nucleus of proliferative cells. However, at present little is known on the traffic between the cytosol and the nucleus. Recently, it has been shown that NHERF1 phosphorylation is involved in its cytoplasm nuclear trafficking (22). In contrast, NHERF1 dephosphorylation by the phosphatase of regenerating liver 3 (PRL-3) causes the nucleus export of NHERF1 to the cytoplasm (23).

NHERF1 can be also regulated at the transcriptional level. NHERF1 gene overexpression is associated with: i) the development of acute inflammatory response (24), ii) a protective role against acute injury (25) and iii) the promotion of metastatic tumor behavior (12). The transcription of NHERF1 is regulated by β-catenin. Indeed, Saponaro and colleagues demonstrated that mRNA and protein levels of NHERF1 expression are negatively regulated by oncogenic β-catenin signalling in colorectal cell lines harboring different Wnt/β-catenin mutations. In particular, nuclear activated β-catenin can regulate NHERF1 directly through the transcriptional factor TCF4 (26).

## NHERF1 and Cervical Cancer

**Figure 3:**
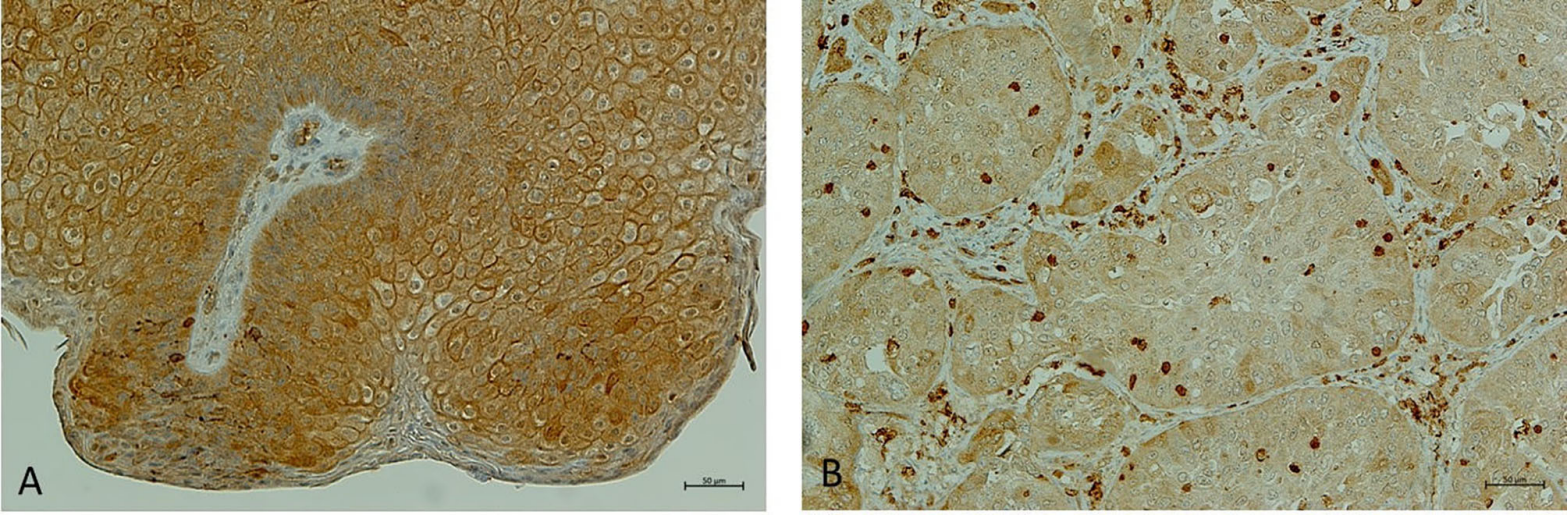
NHERF1 expression in cervical wart. **(A)** Representative image of immunohistochemical staining of high membranous NHERF1 expression in cervical wart; **(B)** Representative image of loss of membranous NHERF1 expression in Cervical squamous cell carcinoma. Original magnification of images ×200. Images were obtained on an Axion Image 2 upright microscope (Zeiss, Oberkochen, Germany) with an Axiocam 512 color camera.

In the last decade, NHERF1 acquired a key role as a prognostic biomarker in cervical cancer (CC) progression (**Figure 3**). The bioinformatics analysis of two GEO datasets and the analysis of validation performed in 31 tissue specimens, demonstrated a down-regulation of NHERF1 expression in cervical cancer samples compared to adjacent normal tissues. Moreover, the down-regulation of NHERF1 was significantly associated with both Wnt signaling and cell proliferation thus suggesting a potential tumor-suppressive role (27). It is likely that NHERF1 inhibits cervical cancer cell proliferation through the downregulation of the actin cross-linking protein α-Actinin-4 (ACTN4). In fact, ACTN4 is functionally implicated in the regulation of β-catenin expression by NHERF1 (27).

One of the mechanisms proposed to explain the down-regulation of NHERF1 in CC is through the interaction of high-risk human papillomavirus (HPV) E6 protein. At COOH terminus, the high-risk HPV-derived E6 oncoprotein presents a PBM domain (PDZ-binding motif) that can bind PDZ proteins, including NHERF1. Importantly, a main functional consequence of E6-induced NHERF-1 degradation is the activation of the PI3K signaling pathway (28). NHERF-1 degradation by E6 appears to be mediated by the protein ubiquitin cell ligase E6-Associated Protein (E6AP). The high-risk HPV E6 proteins bind E6AP and recruit p53 and some PDZ cellular proteins for ubiquitination and degradation by the proteasome. In particular, papillomavirus E6 proteins bind the LQELL sequence of E6AP and stimulate its ubiquitin ligase activity (28). Recently, Drews and colleagues reported NHERF1 degradation by both high and low-risk E6 proteins in association with E6AP, leading to the activation of the Wnt/β-catenin pathway (29). Overall, this supports the E6 oncoprotein role in the activation of the canonical Wnt/β-catenin pathway through NHERF1 degradation in CC.

The contribution of NHERF1 in the regulation of cell motility and invasion in CC has also emerged and several targets identified. For instance, NHERF1 could regulate actin remodeling through ACTN4. In detail, the interaction between NHERF1 and ACTN4 increased ACTN4 ubiquitination and degradation by the proteasome, impacting actin cytoskeleton organization and tumor cell migration and invasion (18). In addition, *in vitro* studies have demonstrated that NHERF1 knockdown is associated with enhanced metalloproteinase 2 (MMP-2) activity, thus further supporting a role for NHERF1 in cancer invasion (30).

In CC samples, NHERF1 has been associated with drug resistance through the regulation of Wnt signaling. A study conducted on patients following cisplatin treatment and patients without cisplatin-treatment, shows a better outcome in patients cisplatin-treated with high NHERF1 expression levels. Similarly, low levels of NHERF1 expression in HeLa cells showed significant cisplatin resistance. These findings indicate that NHERF1 can regulate sensitivity of cervical cancer cells to cisplatin, suggesting the importance to further investigate the molecular mechanisms responsible for cisplatin resistance (31). Previously, other authors described a role for NHERF1 in the multidrug resistance (MDR) process. They reported a closed relationship of NHERF1 with multidrug resistance protein 4 (MPR4), an ATP-binding cassette (ABC)-transporter, in which NHERF1 supported the internalization of this protein. In fact, the down-regulation of NHERF1 in HeLa cells increased the MRP4 expression at the plasma membrane, supposing an inhibition of MRP4 internalization, with consequent growth of drug efflux by this transporter (32).

*Ex vivo* and *in vitro* studies reported a negative correlation of NHERF1 with cell proliferation and epidermal growth factor receptor (EGFR) signaling in CC. Indeed, NHERF1 enacts its tumor suppressive program through the binding of EGFR and inhibition of EGFR-mediated signaling. In CC models, NHERF1 knockdown abolished the inhibition of EGF-induced ERK activation. This highlights the importance of NHERF1 in the regulation of cell proliferation signaling pathways in CC (33).

## NHERF1 and Ovarian Cancer

The biological relevance of NHERF1 in ovarian cancer (OC) pathology has been investigated in several studies. Main findings derive from *ex vivo* and *in vitro* studies that investigated the expression levels of NHERF1 in human samples and defined possible signaling pathways that modulate NHERF1 localization.

*Ex vivo* studies demonstrated an upregulation of NHERF1 in mucinous ovarian carcinomas and ovarian clear cell carcinoma (OCCC). In detail, Tabrizi and colleagues demonstrated an unfavorable prognosis in mucinous ovarian carcinomas with NHERF1 expression (34). In the study of Matsumoto and colleagues, NHERF1 up-regulation has been linked to OCCC recurrence and chemoresistance. The cytoplasmic/nuclear, but not membrane, high expression was significantly observed in recurrent OCCC with respect to primary tumors. Its cytoplasmic overexpression in OCCC cells was associated with a lower susceptibility to cisplatin. Further, the authors demonstrated that NHERF1 was also associated with an increase of poly (ADP-ribose) polymerase 1 (PARP1) expression that is stabilized through the PDZ1 domain of NHERF1. The over-expression of cytoplasmic/nuclear NHERF1 and PARP1 affected patients’ prognosis, being associated with a worse overall and progression-free survival (35).

Kreimann and colleagues investigated the sequence of exons 2 and 3 together with flanking intronic sequences of the *SLC9A3R1* gene that encodes the PDZ2 domain of the NHERF1 protein. In detail, the authors observed the presence of two intronic mutations in the donor splicing site of exon 2 in 8/31 epithelial OC samples analyzed. As this domain is implicated in the binding between NHERF1/β-catenin and NHERF1/E-cadherin, these splicing alterations could potentially modify the localization of these partners (36).

The localization of NHERF1 is regulated by lysophosphatidic acid (LPA). *In vitro*, the treatment of ovarian cancer cells with LPA induces a mobilization of NHERF1 from cytosol to plasma membrane and then into migratory pseudopodia. The translocation is dependent on NHERF1/ERM interaction and the mechanism that underlies this binding is through the ERM phosphorylation (37). In fact, LPA-treatment of OVCAR-3 cells induces a rapid phosphorylation of ERM (cpERM) and the subsequent binding of NHERF1 through its C-terminal ERM-binding domain. Accordingly, the phosphorylation-defective ezrin mutant (Ezrin-T567A) blocked LPA-induced migration of OVCAR-3 cells (37). Overall, the LPA/NHERF1/cpERM axis represents an ideal pathway for molecules able to prevent OC migration and metastasis.

## Conclusions

It has long been clear that NHERF1 exerts an important role in the regulation of signal transduction pathways acting as a scaffold protein. In human tumors, NHERF1 has been found overexpressed in breast cancer, schwannoma, hepatocellular carcinoma and other human tumors. As part of different multiprotein signaling complexes, NHERF1 plays an important role in the propagation of information within the cells. In the last few years, several works have described a double role for NHERF1 in tumorigenesis, according to its subcellular localization. Indeed, NHERF1 displays anti-tumor properties when it is located underneath the plasma membrane; on the contrary, it acts as an oncogene when localized in the cytoplasm or in the nucleus. As multiple levels of regulation exist, translating these data into the clinic is not without complexity.

As the examples in this review highlight, NHERF1 exerts direct biological influences in gynecological tumors. For instance, the overexpression of NHERF1 seems to be required to drive the early phases of cancer onset in different tumor types (**Figure 1**). Moreover, in CC and OC its membrane down-regulation and cytoplasmic over-expression, respectively, trigger signaling pathways linked to cancer progression and drug resistance, among these, the role in the regulation of β-catenin is well described. Overall, these findings suggest possible diagnostic and prognostic clinical implications. In practice, this can be performed in the clinic by immunohistochemistry.

There is also a specific challenge to therapeutically targeting NHERF1. Preliminary findings obtained *in vitro* support the potential therapeutic value of targeted approaches aiming at modulating NHERF1 activity in combination with antagonists of β-catenin (38). Further studies using gynecological models should be designed to demonstrate a defined clinical effect of NHERF1 anti-cancer agents.

## Author Contributions

Concept or design: MS and SS. Acquisition of data: CS and DV. Analysis or interpretation of data: FZ, AT, and CS. Drafting of the article: MS and SS. Critical revision for important intellectual content: AT, DV, MM and FZ. All authors contributed to the article and approved the submitted version.

## Conflict of Interest

The authors declare that the research was conducted in the absence of any commercial or financial relationships that could be construed as a potential conflict of interest.

## Publisher’s Note

All claims expressed in this article are solely those of the authors and do not necessarily represent those of their affiliated organizations, or those of the publisher, the editors and the reviewers. Any product that may be evaluated in this article, or claim that may be made by its manufacturer, is not guaranteed or endorsed by the publisher.
